# Phosphorylation of cGAS by CDK1 impairs self-DNA sensing in mitosis

**DOI:** 10.1038/s41421-020-0162-2

**Published:** 2020-04-28

**Authors:** Li Zhong, Ming-Ming Hu, Li-Jun Bian, Ying Liu, Qiang Chen, Hong-Bing Shu

**Affiliations:** Department of Infectious Diseases, Zhongnan Hospital of Wuhan University, Frontier Science Center for Immunology and Metabolism Medical Research Institute, Wuhan University, Wuhan, 430071 China

**Keywords:** Innate immunity, Cell division

## Abstract

The cyclic GMP-AMP synthase (cGAS) is a widely used DNA sensor, which detects cytosolic DNA species without a preference of self or non-self microbial DNA in interphase to initiate innate immune response. How cGAS is regulated to avoid self-DNA sensing upon nuclear envelope breakdown (NEBD) during mitosis remains enigmatic. Here we show that cGAS is mostly localized in the cytoplasm in interphase and rapidly translocated to chromosomes upon NEBD in mitosis. The major mitotic kinase CDK1-cyclin B complex phosphorylates human cGAS at S305 or mouse cGAS at S291, which inhibits its ability to synthesize cGAMP upon mitotic entry. The type 1 phosphatase PP1 dephosphorylates cGAS upon mitotic exit to enable its DNA sensing ability. Our findings reveal a mechanism on how the DNA sensor cGAS is post-translationally regulated by cell cycle-dependent enzymes to ensure its proper activation for host defense of cytosolic DNA in interphase and inert to self-DNA in mitosis.

## Introduction

Microbial DNA represents a key pathogen-associated molecular pattern (PAMP) that is sensed by host cells during infection^1,2^. Accurate recognition and elimination of foreign DNA while insulating self-DNA from host defense is essential for most organisms. Mechanistically, self-DNA sensing can be prevented by compartmentalization in which self-DNA is sequestered from the cytosol by nuclear envelope or other organelle membranes^1,3^. However, the nuclear envelope dissembles at mitotic entry in vertebrate cells^4^, and the expose of self-DNA to cytosolic sensors raises a question on how mitotic cells restrain innate immunity to avert immune damage.

The cyclic GMP-AMP synthase cGAS is a well-known cytosolic DNA sensor that detects the presence of invaded microbial DNA or self-DNA released into cytoplasm under genomic or mitochondrial stress and senescence^5–10^. Upon binding to DNA, cGAS utilizes GTP and ATP as substrates to synthesize the second messenger 2ʹ,3ʹ-cyclic GMP-AMP (cGAMP), which in turn binds to the ER-located adaptor protein MITA (mediator of IRF3 activation), also called STING (stimulator of IFN genes), to activate downstream transcription factors^11,12^, leading to induction of downstream effector genes such as type I interferons (IFNs) and proinflammatory cytokines. It has been shown that nucleosomes and chromosome-derived micronuclei have decreased abilities to activate cGAS to synthesize cGAMP^13,14^. Since cGAMP is very stable and potent in inducing innate immune response^15^, even low cGAS activity may cause over-reactive immune response upon NEBD. The mechanisms on how cGAS is kept inactive in mitotic cells remain enigmatic.

Post-translational modifications play important roles in regulation of various cellular processes including innate immune response and mitosis. Mitotic progression is precisely controlled by dynamic activation and inactivation of protein kinases and opposing phosphatases, which regulates the phosphorylation states of thousands of proteins^16–18^. Among all kinases, cyclin-dependent kinase 1 (CDK1), which is associated with its regulatory subunit cyclin B, plays a central role in mitotic entry and progression^16^. On the other hand, type 1 (PP1) and type 2A (PP2A) phosphatases play important roles in dephosphorylation of mitotic proteins and mitotic exit to interphase^19,20^.

In this study, we found that cGAS was mostly localized in the cytoplasm in interphase and rapidly translocated to chromosomes upon NEBD in mitosis. The CDK1-cyclin B complex phosphorylates human cGAS (hcGAS) at S305 or murine cGAS (mcGAS) at S291, which inhibits its ability to synthesize cGAMP in mitotic cells. On the other hand, PP1 dephosphorylates cGAS upon mitotic exit to enable its DNA sensing ability. Our findings reveal that the cytosolic DNA sensor cGAS is post-translationally regulated by cell cycle-dependent enzymes to ensure its proper activation for host defense of microbial or aberrant-located self-DNA in interphase and inert to self-DNA during mitosis.

## Results

### Cell cycle-dependent localization of cGAS

**Fig. 1:**
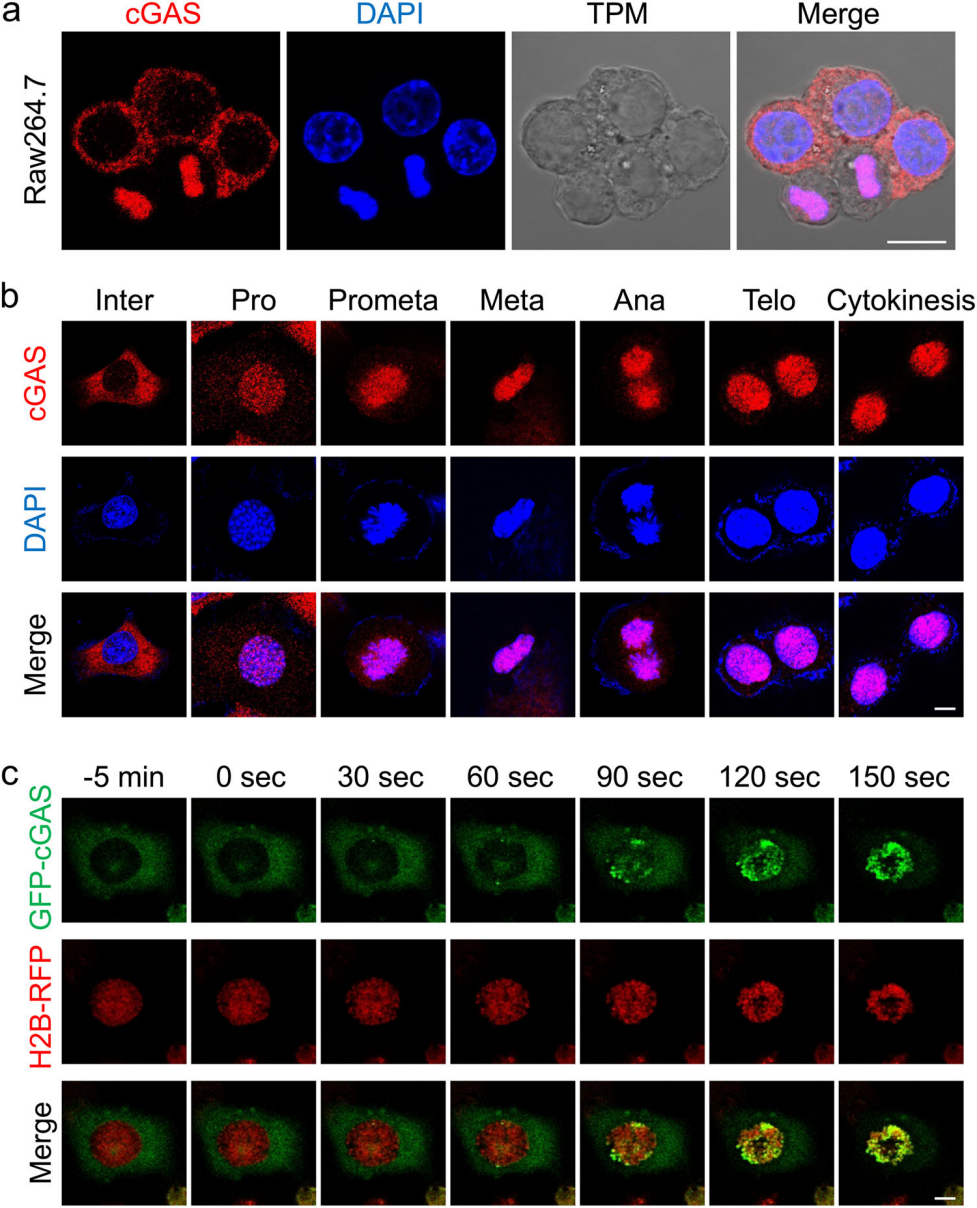
Cell cycle-dependent localization of cGAS. **a** Cellular localization of cGAS in Raw264.7 cells. Raw264.7 cells were fixed and stained with anti-cGAS and DAPI. **b** cGAS is associated with chromosomes in different phases of mitosis. MLF cells were fixed and stained with anti-cGAS and DAPI before confocal microscopy. Inter, interphase; Pro, prophase; Prometa, prometaphase; Meta, metaphase; Ana, anaphase; Telo, telophase. **c** cGAS is associated with chromosomes upon NEBD in living cells. The GFP-cGAS plasmid was transfected into H2B-RFP-expressing HeLa cells for 20 h before live confocal microscopy. Scale bar, 10 μm. Data shown are representative of at least two biological repeats.

To investigate cell cycle-dependent changes of cGAS function, we firstly examined its cellular localization in different phases of the cell cycle. Immunofluorescent staining indicated that endogenous cGAS was mostly localized in the plasma membrane or cytoplasm with minimal level in the nucleus in interphase cells, whereas it was mostly associated with chromosomes in mitotic cells (Fig. 1a, b). Further immunofluorescent staining experiments indicated that endogenous cGAS was associated with chromosomes from prophase to telophase (Fig. 1b). Live cell imaging of HeLa cells transfected with GFP-tagged cGAS indicated that cGAS was promptly translocated to chromosomes upon NEBD at the entry of mitosis (Fig. 1c and Supplementary Movie S1). These results suggest that the localization of cGAS oscillates during cell cycle and cGAS is associated with chromosomes in mitotic cells.

### cGAS inactivation causes unresponsiveness to DNA-triggered innate immunity in mitotic cells

**Fig. 2:**
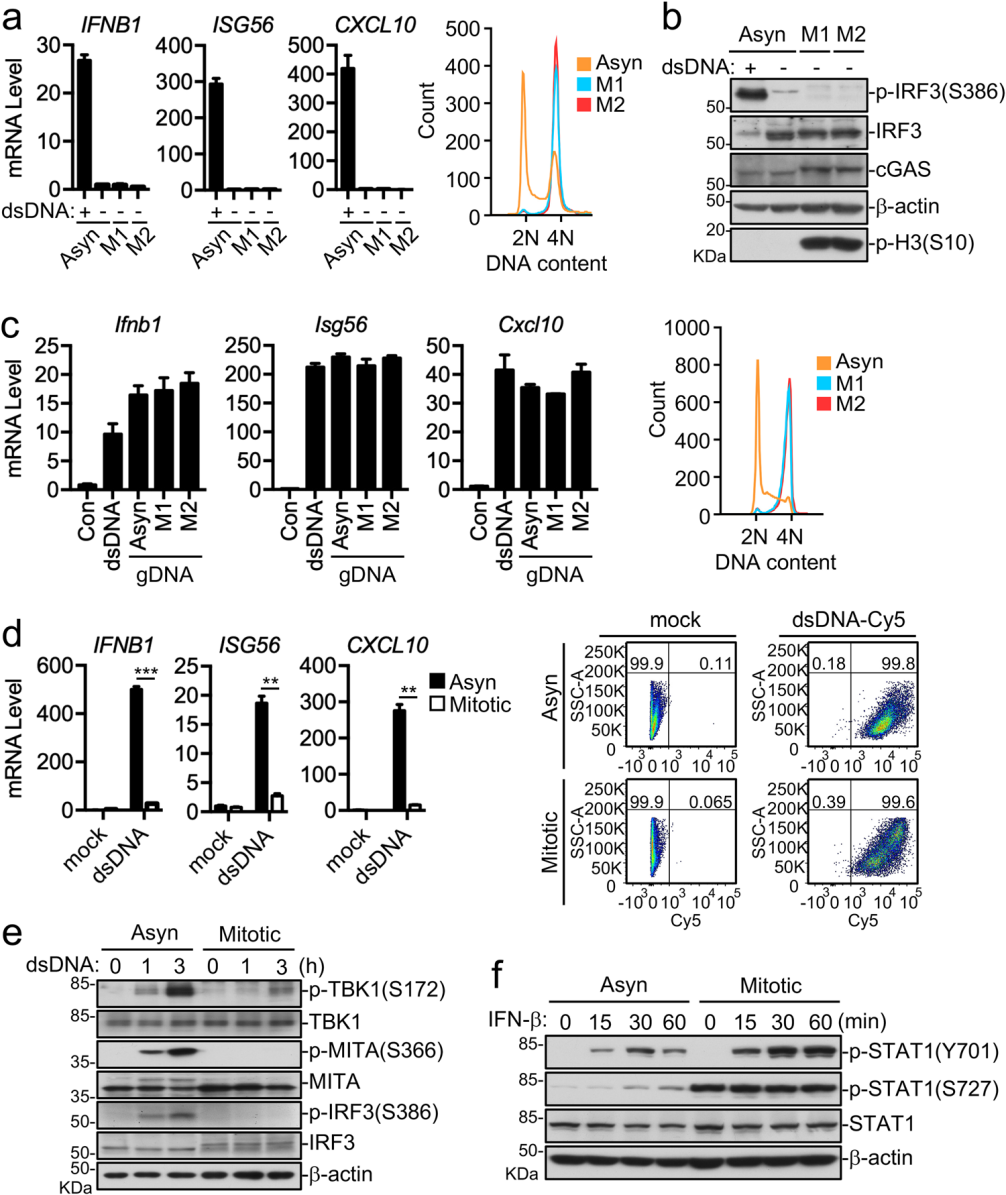
cGAS inactivation causes unresponsiveness to DNA-triggered innate immunity in mitotic cells. **a**, **b** Chromosome-bound cGAS does not activate the IFN response. HT1080 cells were asynchronized (Asyn) or synchronized with nocodazole (M1) or paclitaxel (M2) before qPCR analysis (left) or FASC analysis (right) (**a**), and immunoblotting analysis (**b**). The dsDNA HSV120 (**a**) or HT-DNA (**b**)-transfected asynchronous cells were used as positive control. **c** Activation of cGAS by mitotic DNA. Genomic DNAs (gDNA) derived from asynchronized (Asyn), nocodazole (M1) or paclitaxel (M2) synchronized HeLa cells were transfected into MLF cells before qPCR analysis. The dsDNA DNA90 was used as a positive control. Data shown are mean ± SD, *n* = 3. **d**, **e** cGAS-MITA signaling is impaired in mitotic cells. HT1080 cells asynchronized (Asyn) or synchronized with paclitaxel (Mitotic) were mock-transfected or transfected with the dsDNA DNA90 (**d**) or HSV120 (**e**) before qPCR (**d**) and immunoblotting analysis (**e**). HSV60-Cy5 was used to analysis the transfection efficiency by FACS (**d**). **P < 0.01; ***P < 0.001 (Student’s *t*-test, unpaired, two-tailed). **f** IFN-β-induced STAT1 Y701 phosphorylation in mitosis. HT1080 cells asynchronized (Asyn) or synchronized by paclitaxel (Mitotic) were left untreated or treated with IFN-β for the indicated times before immunoblotting analysis. Data shown are representative of three (**a**–**c**) or two (**d**–**f**) biological repeats. Data shown in (**a**), (**c**), and (**d**) are mean ± S.D. of one representative experiment performed in triplet.

As cGAS binds to chromosomes during mitosis, we determined whether the chromosome-bound cGAS mediates innate immune response in mitotic cells. To do this, we synchronized HT1080 cells by nocodazole or paclitaxel and collected mitotic cells by the shake-off method. qPCR experiments indicated that transcription of downstream genes including *IFNB1*, *ISG56*, and *CXCL10* was barely detectable in both mitotic and asynchronous cells (Fig. 2a). Immunoblotting experiments indicated that phosphorylation of IRF3 S386, which is a hallmark of cGAS-mediated activation of downstream events^21^, was also barely detectable in both mitotic and asynchronous cells (Fig. 2b). In these experiments, transfected dsDNA potently induced transcription of the downstream effector genes and IRF3 S386 phosphorylation (Fig. 2a, b). These results suggest that cGAS-mediated innate immune response is inactive even though cGAS is associated with chromosomes in mitotic cells.

We next investigated the mechanisms on why chromosome-bound cGAS does not initiate innate immune response in mitotic cells. We firstly determined whether genomic DNA from mitotic cells could trigger innate immune signaling. We transfected murine lung fibroblasts (MLFs) with synthetic dsDNA and genomic DNA derived from asychronized and nocodazole or paclitaxel synchronized HeLa cells and then examined mRNA levels of downstream effector genes. The results indicated that genomic DNA derived from asynchronous and mitotic cells induced transcription of *Ifnb1*, *Isg56*, and *Cxcl10* genes to similar levels, which was also comparable to that induced by synthetic dsDNA (Fig. 2c). These results suggest that genomic DNA of mitotic cells is equally capable of inducing innate immune response.

We next transfected synthetic dsDNA into asynchronous and mitotic HT1080 cells, and measured the mRNA levels of *IFNB1, ISG56 CXCL10* genes. The results indicated that dsDNA-induced transcription of downstream effector genes in asynchronous but not mitotic cells (Fig. 2d). In addition, transfected dsDNA-induced phosphorylation of MITA S366, TBK1 S172, and IRF3 S386, which are hallmarks of activation of cGAS downstream components, in asynchronous but not mitotic cells (Fig. 2e). These results suggest that the cGAS-mediated pathways do not respond to dsDNA stimulation in mitotic cells. Interestingly, the downstream cytokine IFN-β-induced STAT1 Y701 phosphorylation was increased in mitotic cells in comparison to asynchronous cells (Fig. 2f). These results suggest that inactivation of cGAS-mediated signaling in mitotic cells is not a generic character of cellular signaling events.

### Phosphorylation of hcGAS S305 or mcGAS S291 causes its inactivation in mitosis

**Fig. 3:**
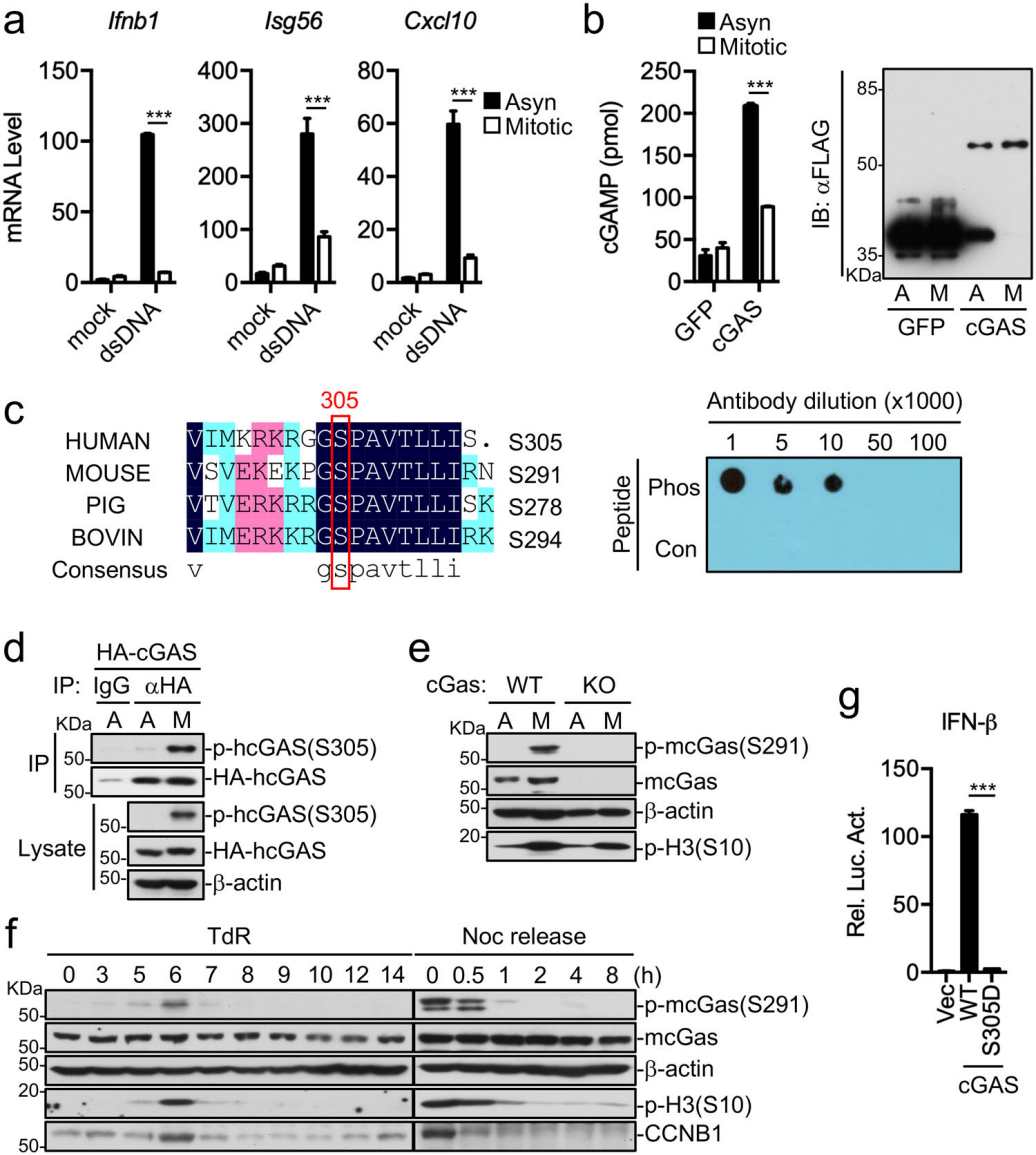
Phosphorylation of cGAS S305 causes its inactivation in mitosis. **a** dsDNA-induced production of cGAMP is impaired in mitotic cells. Asynchronized (Asyn) or synchronized (Mitotic) HT1080 cells were mock-transfected or transfected the dsDNA DNA90 for 4 h and then cell extracts containing cGAMP were delivered to digitonin-permeabilized Raw264.7 cells for 4 h before qPCR analysis of mRNA levels of the indicated genes. **b** Mitotic cGAS has decreased enzymatic activity. cGAS purified from asynchronized (Asyn) or synchronized (Mitotic) HT1080 cells expressing FLAG-cGAS was subjected to in vitro cGAMP synthesis assay. FLAG-GFP was used as a negative control. **c** Preparation of a mcGAS S291 phosphorylation antibody. Left: Sequence alignment of cGAS from the indicated species. The sequences are corresponding to aa284-300 of mcGAS. Right: A phospho-S291 mcGAS antibody specifically recognized the phosphorylated peptide. Synthetic peptides of phosphorylated (Phos) or control (Con) mcGas _284_VEKEKPGSPAVTLLIRN_300_ were used for dot blots. **d**, **e** cGAS is phosphorylated at hcGAS S305 or mcGAS S291 in mitotic cells. HA-cGAS stably-expressing HT1080 cells were asychronized (A) or synchronized at mitosis (M). The cell lysates were co-immunoprecipitated with anti-HA before immunoblotting analysis with the indicated antibodies (**d**). *cGas*^*+/+*^ and *cGas*^*−/−*^ L929 cells were treated with nocodazole (300 nM) for 14 h before immunoblotting analysis with the indicated antibodies (**e**). **f** Cell cycle-dependent regulation of cGAS phosphorylation. L929 cells were arrested at G1/S transition with double-thymidine blockade, followed by release for different times (TdR) or were arrested at mitosis using nocodazole blockade, followed by shake-off and release for different times (Noc release). Cells were collected and subjected to immunoblotting analysis with the indicated antibodies. p-H3 (Histone H3 phospho S10) and cyclin B was used as a mitotic marker. **g** cGAS phosphorylation mimic mutant is inactive. MITA stably-expressing HEK293 cells were transfected with the indicated plasmids for 24 h before luciferase assays. ***P < 0.001 (Student’s *t*-test, unpaired, two-tailed). Data shown are representative of three (**a**, **d**) or two (**b**, **e**–**g**) biological repeats. Data shown in (**a**), (**b**), and (**g**) are mean ± S.D. of one representative experiment performed in triplet.

Since the transfected dsDNA HSV120 failed to induce phosphorylation of MITA S366 in mitotic cells (Fig. 2e), we hypothesized that MITA or it is upstream dsDNA sensor cGAS is inactivated in mitotic cells. We examined cGAMP production upon transfection of the synthetic dsDNA DNA90 into asynchronous or mitotic H1080 cells. The results indicated that dsDNA-transfected mitotic cells produced lower amounts of cGAMP in comparison to dsDNA-transfected asynchronous cells (Fig. 3a). In vitro experiments indicated that cGAS purified from mitotic cells had lower activity to synthesize cGAMP in comparison to cGAS purified from asynchronous cells (Fig. 3b). These results suggest that cGAS in mitotic cells is inert for dsDNA.

It has been demonstrated that various nuclear proteins and metabolic enzymes are inactivated by phosphorylation in mitotic cells^22^. Since we routinely observed that cGAS in mitotic cells was shifted to slightly higher molecular weight band in immunoblotting experiments (Fig. 2b), we tested the hypothesis that phosphorylation of cGAS during mitosis leads to its inability to sense dsDNA. Previously, it has been shown that AKT-mediated phosphorylation of cGAS S305 suppresses its enzymatic activity^23^. We generated an antibody that could specifically recognize mcGAS S291-phosphorylated epitope, which is also highly conserved among various mammals (Fig. 3c). Co-immunoprecipitation and immunoblotting experiments indicated that hcGAS S305 (Fig. 3d) or mcGAS S291 (Fig. 3e) were markedly phosphorylated in mitotic but barely in asynchronous cells. The low basal phosphorylation of cGAS in asynchronous cells is probably due to exist of a small fraction of mitotic cells. Flow cytometry analysis confirmed that phosphorylation of mcGAS S291 occurred in mitotic cells (Supplementary Fig. S1). Double-thymidine block and nocodazole release experiments indicated phosphorylation of mcGAS S291 was tightly correlated with cyclin B level and phosphorylation of histone H3 S10 (Fig. 3f), which are tightly regulated during cell cycle^24^. These results indicated that mcGAS S291 was phosphorylated upon entry to mitosis and dephosphorylated upon exit of mitosis. On the other hand, cGAS-deficiency did not affect cell cycle progression (Supplementary Fig. S2). To determine the functional significance of cell cycle-dependent phosphorylation of cGAS, we performed mutagenesis study. Mutation of hcGAS S305 to aspartic acid (S305D), which mimics its phosphorylation, abolished its ability to activate the IFN-β reporter (Fig. 3g). These results suggest that phosphorylation of hcGAS S305 impairs its activity. DNA pull-down experiments indicated that wild-type hcGAS and hcGAS(S305D) bound to the dsDNA HSV120 to comparable levels (Supplementary Fig. S3a). Structure analysis indicated that S305 is localized in the catalytic pocket of hcGAS (Supplementary Fig. S3b). The simplest explanation for these data is that hcGAS S305 phosphorylation does not affect its dsDNA-binding ability, but impairs its substrate binding or catalytic reaction.

Since S305 is located in the nuclear localization sequence (_295_DVIMKRKRGGS_305_) of hcGAS^25^, we investigated whether phosphorylation of S305 is required for its chromosomal localization during mitosis. Immunofluorescent staining experiments indicated that hcGAS(S305A) was localized to chromosomes in mitotic cells similarly as wild-type hcGAS or hcGAS(S305D) (Supplementary Fig. S3c). These results suggest that phosphorylation of hcGAS S305 is not required for its chromosomal localization in mitotic cells.

### The CDK1-cyclin B complex mediates cGAS phosphorylation and inactivation in mitotic cells

**Fig. 4:**
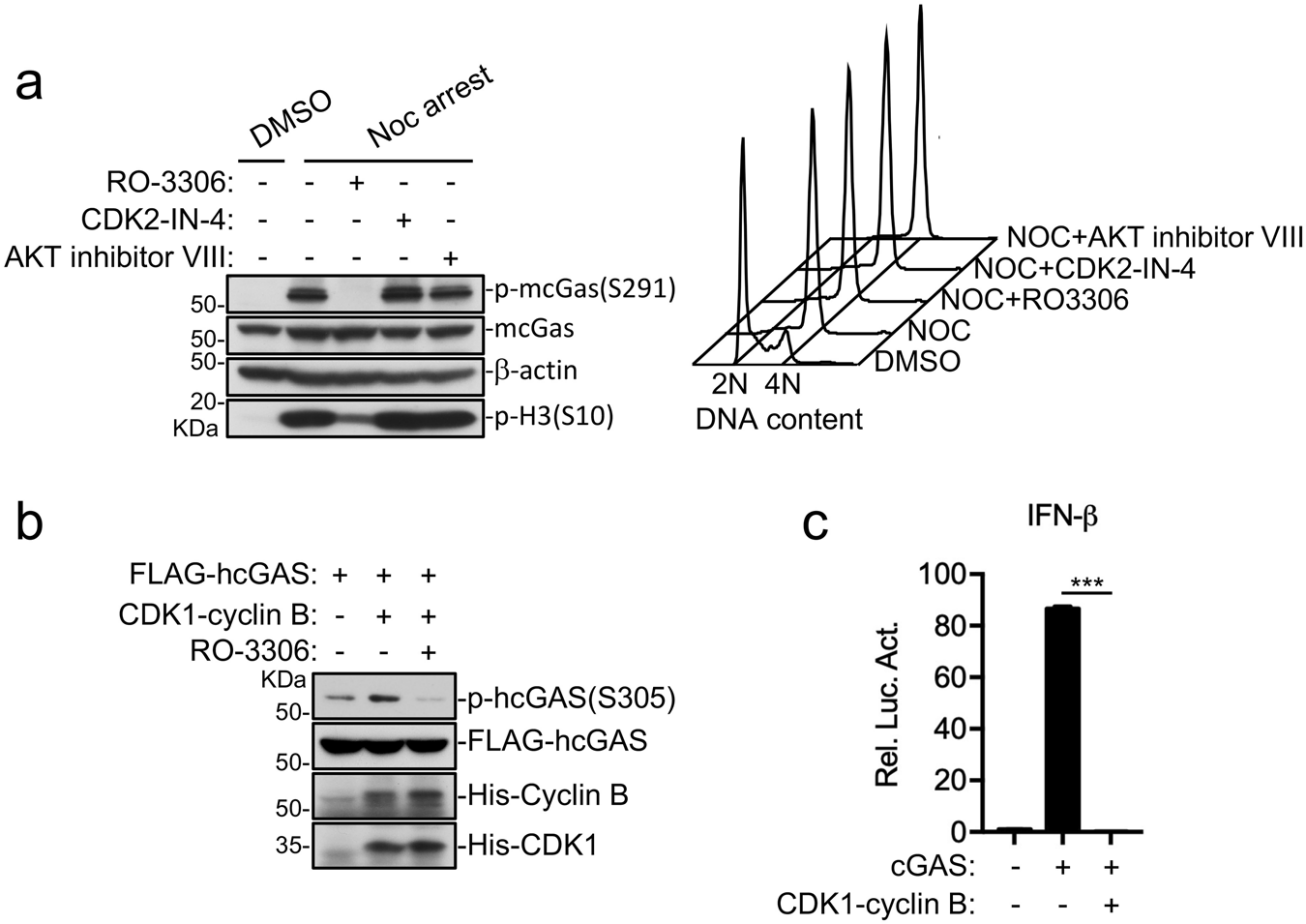
The CDK1-cyclin B complex accounts for cGAS phosphorylation and inactivation in mitotic cells. **a** CDK1 but not AKT or CDK2 inhibitor abolishes mcGAS S291 phosphorylation. Raw264.7 cells were arrested at mitosis with nocodazole (Noc arrest) and then treated with the indicated kinase inhibitors (10 μM) for 15 min before immunoblotting or FACS analysis. **b** cGAS is a direct substrate of the CDK1-cyclin B complex. In vitro kinase assays were performed with recombinant CDK1-Cyclin B proteins and FLAG-cGAS immunoaffinity-purified from THP1 cells stably-expressing FLAG-cGAS in the presence or absence of RO-3306 (10 μM). The reactions were then analyzed by immunoblotting analysis with the indicated antibodies. **c** The CDK1-cylin B1 complex impairs cGAS activation. MITA stably-expressing HEK293 cells were transfected with the indicated plasmids for 24 h before luciferase assays. ***P < 0.001 (Student’s *t*-test, unpaired, two-tailed). Data shown are mean ± S.D. of one representative experiment performed in triplet. Data are representative of three biological repeats.

We next determined the kinase(s) responsible for cGAS phosphorylation in mitotic cells. Sequence analysis with Scansite 4.0 (https://scansite4.mit.edu/) indicated that CDK1 and CDK5 are potential kinases for phosphorylation of hcGAS S305 or mcGAS S291. Unlike other classical CDKs, CDK5 does not directly regulate cell cycle progression and its activity is thought to be restricted in terminally differentiated cells^26^. Therefore, we investigated whether CDK1 is responsible for phosphorylating cGAS in mitotic cells. We found that treatment of nocodazole-arrested mitotic cells with the CDK1 inhibitor RO-3306 for 15 min abolished mcGAS S291 phosphorylation, whereas the AKT inhibitor VIII and the CDK2 inhibitor CDK2-IN-4 had no marked effects (Fig. 4a). In these experiments, transient treatment with the inhibitors did not affect mitotic status of the cells (Fig. 4a). We further evaluated the ability of CDK1 to phosphorylate cGAS by in vitro kinase assay using purified CDK1-cyclin B complex. The results indicated that hcGAS S305 was phosphorylated by the CDK1-cyclin B complex, which was inhibited by the CDK1 inhibitor RO-3306 (Fig. 4b). Overexpression of CDK1 and cyclin B1 abolished cGAS-mediated activation of the IFN-β promoter in MITA-expressing HEK293 cells (Fig. 4c). Collectively, our results suggest that the CDK1-cyclin B1 complex phosphorylates hcGAS at S305 to prevent its activation during mitosis.

### Inhibition of cGAS phosphorylation causes its activation in mitotic cells

**Fig. 5:**
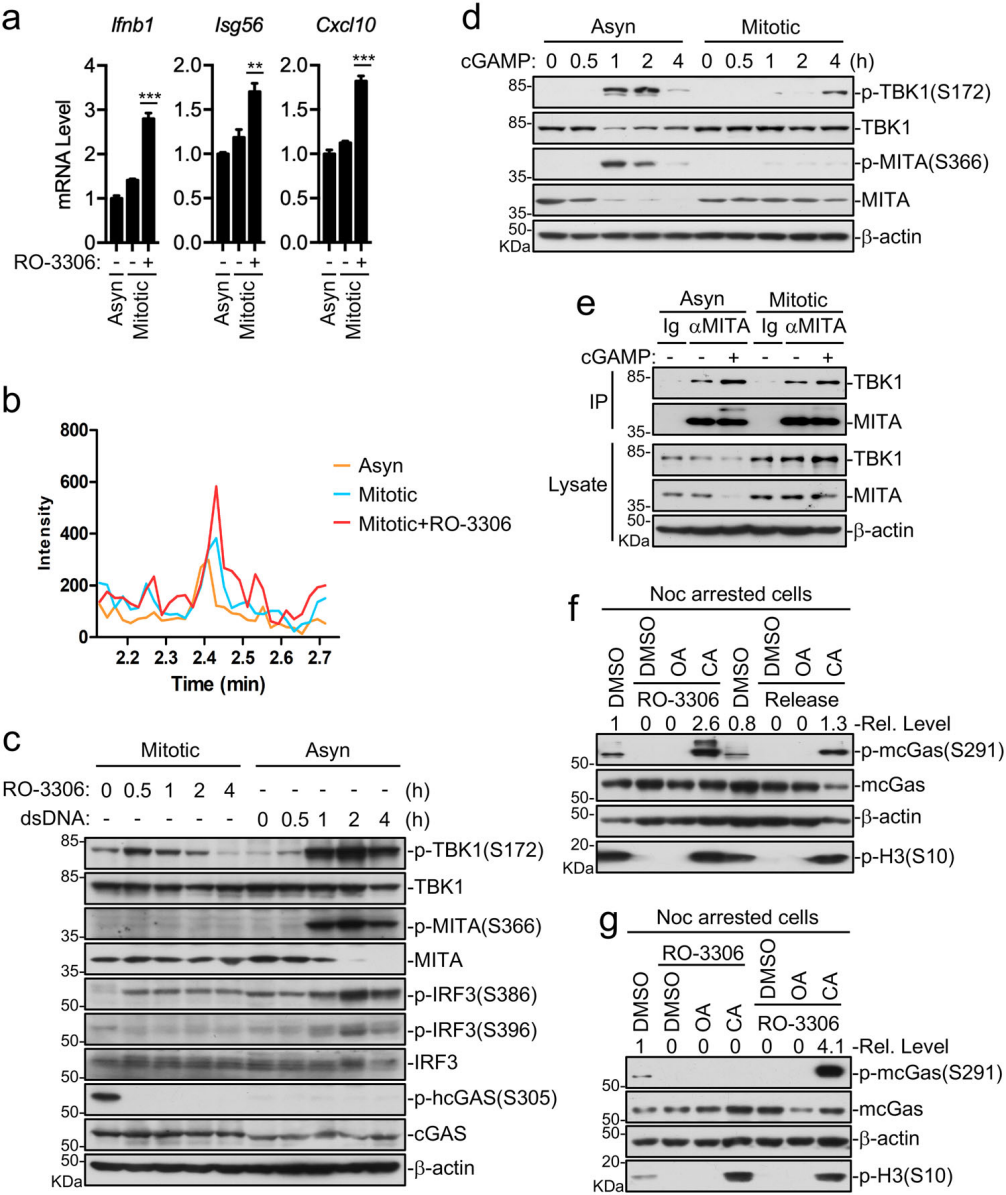
Multiple mechanisms are involved in inhibition of cGAS-mediated pathways in mitotic cells. **a**, **b** CDK1 inhibition partially restores cGAMP production. Asynchronized (Asyn) or synchronized (Mitotic) HT1080 cells were left untreated or treated with RO-3306 (10 μM) for 30 min, and then cell extracts containing cGAMP were delivered to digitonin-permeabilized Raw264.7 cells for 4 h before qPCR analysis for mRNA levels of the indicated genes (**a**), or were directly analyzed by mass spectrometry (**b**). **P < 0.01, ***P < 0.001 (Student’s *t*-test, unpaired, two-tailed). Data shown are mean ± S.D. of one representative experiment performed in triplet (**a**). **c** Effects of CDK1 inhibition on activation of downstream components of cGAS in mitotic cells. Synchronized (Mitotic) HT1080 cells were left untreated or treated with RO-3306 (10 μM) for the indicated times before immunoblotting analysis with the indicated antibodies. Asynchronized (Asyn) HT1080 cells transfected with HT-DNA were used as a positive control. **d** Inhibition of downstream components of cGAS in mitosis. HT1080 cells asynchronized (Asyn) or synchronized by paclitaxel (Mitotic) were left mock-transfected or transfected with cGAMP for the indicated times before immunoblotting analysis with the indicated antibodies. **e** cGAMP-induced recruitment of TBK1 to MITA is barely affected in mitotic cells. Asynchronized (Asyn) or synchronized (Mitotic) HT1080 cells were left untreated or treated with cGAMP for 2 h followed by co-immunoprecipitation and immunoblotting analysis. **f** CA but not OA restores mcGas phosphorylation at S291. Raw264.7 cells were synchronized by nocodazole, pretreated with the phosphatase inhibitors (100 nM) for 30 min, followed by RO-3306 treatment (10 μM) for 30 min or release into fresh medium for 2 h before immunoblotting analysis. **g** CDK1 and PP1 coordinately regulate cGAS phosphorylation during mitosis. Synchronized (Mitotic) Raw264.7 cells were pretreated with RO-3306 (10 μM) for 30 min then treated with CA or OA (100 nM) for 30 min, or were pretreated with CA or OA (100 nM) for 30 min and then treated with RO-3306 (10 μM) for 30 min. The cells were then analyzed by immunoblots with the indicated antibodies. Data shown are representative of three (**a**) or two (**d**–**g**) biological repeats.

Since phosphorylation of cGAS S305 by CDK1 in mitotic cells inhibits its activity, we next determined whether inhibition of CDK1 activity could activate cGAS. We found that CDK1 inhibitor RO-3306 increased cGAMP production in mitotic cells, suggesting that inhibition of CDK1-mediated cGAS phosphorylation leads to its activation (Fig. 5a, b). Previously, it has been shown that binding of cGAMP to MITA results in recruitment and autophosphorylation of TBK1 at S172, which then phosphorylates MITA at S366 and further recruits IRF3 to this complex, leading to subsequent phosphorylation of IRF3 S386/S396 by TBK1 and induction of downstream antiviral genes^27,28^. Treatment of mitotic cells with both RO-3306 (Fig. 5c) and cGAMP (Fig. 5d) failed to induce MITA S366 or IRF3 S386/S396 but weakly induced TBK1 S172 phosphorylation. Since both RO-3306 and cGAMP weakly activated TBK1 in mitotic cells, we determined whether TBK1 is recruited to MITA upon cGAMP stimulation in mitotic cells. The results indicated that similar to interphase cells, cGAMP stimulation increased the recruitment of TBK1 to MITA in mitotic cells (Fig. 5e). The simplest explanation for these observations is that cGAMP-induced recruitment of TBK1 to MITA and subsequent self-activation of TBK1 are not affected in mitotic cells. However, phosphorylation of MITA and IRF3 by TBK1 is inhibited in mitotic cells by unknown mechanisms.

### PP1 dephosphorylates cGAS upon mitotic exit

**Fig. 6:**
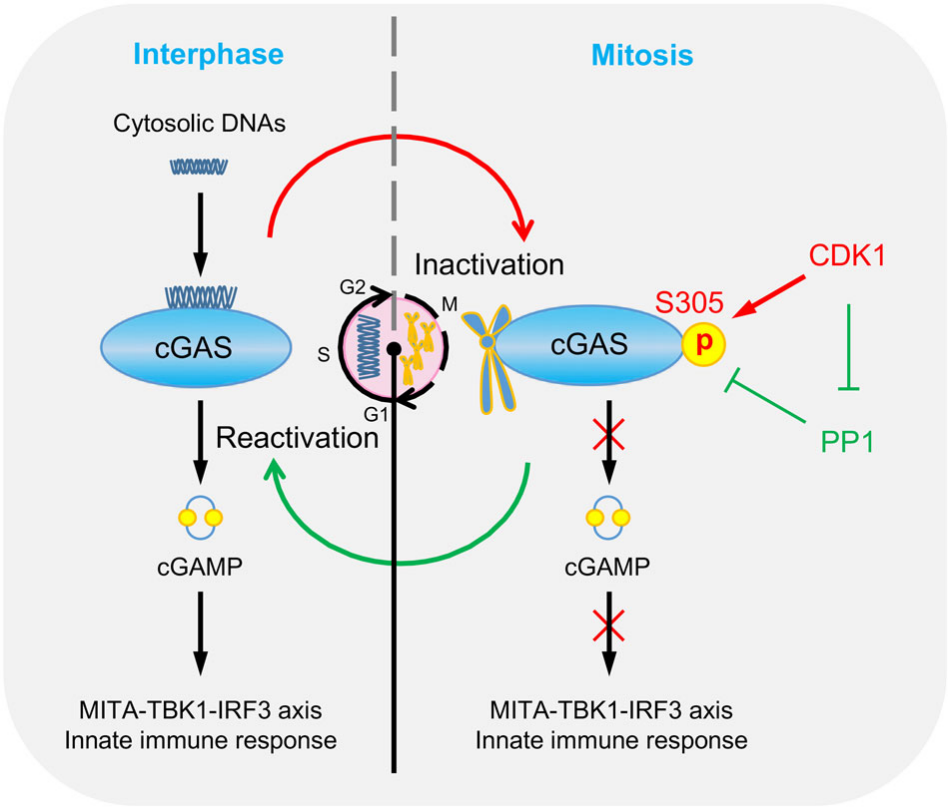
Inactivation of cGAS in mitosis. Upon entry to mitosis, cGAS is translocated to chromosomes. The CDK1-cyclin B kinase complex phosphorylates hcGAS at S305 or mcGAS at S291, leading to its inability to synthesize cGAMP in mitotic cells. Upon mitotic exit, PP1 mediates cGAS dephosphorylation which enables its ability to scrutinize the presence of microbial or aberrant-located self-DNA in interphase.

Since cGAS is dephosphorylated upon mitotic exit or CDK1 inhibition (Figs. 3f and 4a), we investigated whether the dephosphorylation is mediated by a phosphatase involved in mitotic exit. Both Calyculin A (CA) and Okadaic acid (OA) are potent inhibitors for PP2A, PP4, and PP5. However, only CA but not OA can potently inhibit PP1^29^. To determine which phosphatase is responsible for dephosphorylating cGAS upon mitotic exit, mitotic arrested cells were pretreated with either CA or OA, then further treated with the CDK1 inhibitor RO-3306 or released from mitotic arrest by removal of nocodazole from the medium. Immunoblotting experiments showed that CA but not OA inhibited mcGAS S291 dephosphorylation (Fig. 5f). These results suggest that PP1 mediates the dephosphorylation of cGAS upon mitotic exit. It has been reported that CDK1 inhibits the opposite phosphatases to achieve maximal phosphorylation of substrates in early mitosis, therefore the decreased phosphorylation level of cGAS by CDK1 inhibition may be caused by reactivation of PP1. Combination of kinase and phosphatase inhibition in a timely manner, we found that CA treatment did not restore mcGAS S291 phosphorylation in mitotic cells that were pretreated with the CDK1 inhibitor RO-3306 (Fig. 5g). These data suggest that inhibition of CDK1 activity leads to PP1 reactivation, which causes the dephosphorylation of mcGAS at S291. However, CA pretreatment antagonized dephosphorylation of mcGAS at S291 induced by CDK1 inhibition (Fig. 5f, g). Taken together, these findings suggest that CDK1 phosphorylates both cGAS and PP1 to inhibit their activities and to achieve maximal inhibition of cGAS in mitotic cells. On the other hand, PP1 reactivation mediates dephosphorylation of cGAS upon mitotic exit, which enables its ability to scrutinize the presence of microbial or aberrant-located self-DNA in interphase cells (Fig. 6).

## Discussion

Higher eukaryotic cells adopt ‘open’ mitosis to ensure precise division of its genome into two daughter cells. The fact that the cytosolic DNA sensor cGAS translocate to chromosomes upon NEBD poses an important question on why cGAS is inert to sense self-DNA in mitosis.

In this study, we found that cGAS purified from mitotic cells lost its ability to synthesize cGAMP, suggesting that cGAS is not capable of sensing self-DNA in mitotic cells. We further showed that hcGAS S305 or mcGAS S291 was phosphorylated in mitotic cells. Purified CDK1-cyclin B complex phosphorylated hcGAS S305 in vitro, indicating cGAS is a direct substrate of CDK1. Mutation of hcGAS S305 to D, which mimics its phosphorylation, abolished its ability to activate downstream signaling. In addition, overexpression of the CDK1-cyclin B complex impaired cGAS-mediated activation of the IFN-β promoter. These findings suggest that the CDK1-cyclin B kinase complex is responsible for cGAS phosphorylation and inactivation in mitotic cells. Except for S305, the residues S201, S272, S333, and S435 of hcGAS are conserved among various mammals and predicted as potential phosphorylation sites targeted by CDK1. Whether these residues are indeed phosphorylated by CDK1 and the functional significance of the potential phosphorylation requires further investigation.

Upon exit of mitosis, timely reactivation of cGAS is essential for host defense of microbial or aberrant-located self-DNA in interphase cells. Our results indicated that the phosphatase PP1 dephosphorylated cGAS upon mitotic exit, therefore enabling it to scrutinize the presence of cytosolic DNA. Taken together, these results suggest that cGAS is regulated by cell cycle-dependent enzymes to ensure its proper inactivation and reactivation (Fig. 6). Both CDK1 and PP1 play important roles in regulation of mitotic progression. By controlling cGAS activity through these key components of the mitotic machinery, mammalian cells can precisely coordinate innate immune response with mitosis.

It has been previously shown that AKT can phosphorylate hcGAS S305 as CDK1 does^23^. Our results indicated that the AKT inhibitor VIII failed to inhibit mcGAS S291 phosphorylation, whereas the CDK1 inhibitor RO-3306 abolished mcGAS S291 phosphorylation in mitotic cells. These results suggest that CDK1 is the major kinase responsible for mcGAS S291 phosphorylation during mitosis. Why AKT does not contribute to this phosphorylation in mitotic cells is unknown.

cGAS detects cytosolic DNA species without a preference for self or non-self-DNA^3^. It is intriguing to understand the physiological significance on why cGAS predominantly translocates to chromosomes in mitotic cells. Micronuclei are formed from mis-segregation of chromosomes during mitosis^13^. Recently, cGAS was found to promote micronuclei formation and cell death in respond to DNA damage^30^. It is possible that the chromosomal localization of cGAS in mitotic cells contributes to eradication of cells with damaged genomes.

Our results indicated that inhibition of CDK1 by RO-3306 only partially restored cGAS activity. It is possible that phosphorylation of cGAS by CDK1 is not the only factor responsible for cGAS inactivation in mitosis. As cGAS binds to self-DNA in mitotic cells, even very low cGAS activity may cause severe immune damage to host cells. Therefore, multiple mechanisms may be responsible for completely inactivating cGAS. Immunoblotting experiments indicated that the molecular weight of cGAS in mitotic cells is still shifted to a higher molecular weight band in the existence of CDK1 inhibitors (Fig. 5c), suggesting that other post-translation modifications of cGAS exist during mitosis. Numerous studies have demonstrated that post-translation modifications, including phosphorylation, ubiquitination, sumoylation and glutamylation, play important roles in regulation of cGAS activity^31–34^. Whether other post-translational modifications are involved in regulation of cGAS activity during mitosis needs further investigation. Except for post-translational modifications, it has been reported that co-receptors are important for cGAS activation in interphase cells^2,35,36^. Whether suppressors of cGAS are involved in the inactivation of cGAS in mitosis also needs further investigation.

Recently, it has been reported that a fraction of cGAS is constitutively localized in the nucleus of certain types of interphase cells^37,38^. It has been shown that cia-cGAS (circular RNA antagonist for cGAS) can bind nuclear cGAS and inhibits its enzymatic activity in long-term hematopoietic stem cells^37^. It has also been shown that nuclear cGAS is tethered tightly by a salt-resistant interaction which prevents its autoreactivity against self-DNA in cells^39^. It would be interesting to investigate whether these mechanisms are involved in inactivation of cGAS in mitotic cells, and on the other side, whether hcGAS S305 or mcGAS S291 phosphorylation contributes to inactivation of nuclear cGAS in interphase cells.

Our data showed that cGAMP-induced phosphorylation of MITA was inhibited, whereas the recruitment of TBK1 to MITA and its subsequent autoactivation were not affected in mitotic cells. The underlying mechanisms need further investigation. We showed that cGAS lacks the ability to sense DNA in mitotic cells, then how mitotic cells defend DNA virus infection? We noticed that IFN-β-induced STAT1 activation was markedly enhanced in mitotic cells in comparison to asynchronous cells. It is likely that antiviral response of mitotic cells was stimulated by type I IFNs produced by nearby viral infected interphase cells. In conclusion, our findings reveal mechanisms on how cGAS is temporally and spatially regulated during cell division so that it can sense microbial and mis-located self-DNA in the cytosol of interphase cells but inert to self-DNA during mitosis.

## Materials and methods

### Reagents, antibodies, and cells

2ʹ,3ʹ-cGAMP and Lipofectamine 2000 (InvivoGen); polybrene (Millipore); RNAiso Plus (Takara); SYBR Green mix (Bio-Rad); Dual-Specific Luciferase Assay Kit (Promega); nocodazole, digitonin, thymidine, okadaic acid, HT-DNA (Sigma); paclitaxel and RO-3306 (Selleck); puromycin (Thermo); purified CDK1-cyclin B complex and Hoechst33442 (Life Technologies); Malachite Green Phosphate Detection Kit (CST); calyculin A (MCE); DNeasy® Blood & Tissue Kit (Qiagen); recombinant IFN-β (R&D systems) were purchased for the indicated manufacturers.

Mouse monoclonal antibodies against HA (Origene, Cat# TA180128); FLAG (Sigma, F3165) and β-actin (Sigma, A2228); rabbit antibodies against cGAS (Cell Signaling Technology, 66546S/31659S), phosphor-Tyrosine701-STAT1 (Cell Signaling Technology, 9167S), phosphor-Serine727-STAT1(Cell Signaling Technology, 8826S), phosphor-Serine396-IRF3 (Cell Signaling Technology, 4947S), phosphor-Serine366-MITA (Cell Signaling Technology, 50907S), MITA (Cell Signaling Technology, 13647S); phosphor-Serine-TBK1 (Abcam, ab109272), TBK1 (Abcam, ab40647), phosphor-Serine386-IRF3 (Abcam, ab76493); phosphor-Serine10-Histone H3 (Zenbio, Cat No. 301271); IRF3 (Santa Cruz Biotechnology, sc-33641) and STAT1 (Santa Cruz Biotechnology, sc-417) were purchased from the indicated manufacturers. Antisera against phosphor-Serine291-mcGAS were generated by immunizing rabbits with synthetic peptide of mouse cGAS (_284_VEKEKPGSPAVTLLIRN_300_) by ABclonal Technology (Wuhan).

The 293T cells were originally provided by Dr. Gary Johnson (National Jewish Health); THP1 cells were obtained from ATCC; H2B-RFP expressing HeLa cells were provided by Dr. Qiang Chen (Wuhan University); HT1080 and Raw264.7 cells were obtained from CCTCC (China Center for Type Culture Collection); *cGas*^*+/+*^ and *cGas*^*−/−*^ L929 cells were provided by Dr. Jiahuai Han (Xiamen University); and MLFs were prepared as described^40^.

### Cell culture and synchronization

Cells were cultured in DMEM with 10% fetal bovine serum, 100 U/ml penicillin and 100 μg/ml streptomycin. For cell synchronization, HT1080 and HeLa cells were treated with nocodazole (100 nM) or paclitaxel (100 nM) for the indicated times and collected by mitotic shake-off. For double-thymidine block and release experiments, L929 cells were arrested for 16 h with 2.5 mM thymidine with a 12 h release interval or arrested by nocodazole (300 nM). Mitotic cells were collected by the shake-off method. The arrested cells were released into fresh medium to follow cell cycle progression. For inhibitor treatment, Raw264.7 cells were synchronized by nocodazole (150 nM) arrest for 16 h before inhibitor treatment and immunoblotting analysis.

### Constructs

Expression plasmids for HA-, FLAG-, GFP-tagged cGAS, cGAS point mutants and HA-, FLAG-tagged CDK1 were constructed by standard molecular biology techniques. The IFN-β promoter reporter plasmid was previously described^41^. The expression plasmid for cyclin B1 was purchased from Origene.

### DNA oligonucleotides

The sequences of DNA90 and HSV120 were previously described^35^.

### Transfection

For reporter assays, MITA-expressing HEK293 cells were transfected with the indicated plasmids by standard calcium phosphate precipitation method. To ensure that each transfection receives the same amount of total DNA, an empty control plasmid was added to each transfection. To normalize for transfection efficiency, pRL-TK (*Renilla* luciferase) reporter plasmid (0.01 μg) was added to each transfection. Luciferase assays were performed using a Dual-Specific Luciferase Assay Kit. Firefly luciferase activities were normalized on the basis of *Renilla* luciferase activities.

For DNA-induced innate immune response experiments, the isolated genomic DNA or synthetic dsDNA (1 μg/ml) was transfected into MLF cells for 3 h by Lipofectamine 2000 (InvivoGen) followed by qPCR analysis.

### qPCR

Total RNA was isolated for qPCR analysis to measure mRNA abundances of the indicated genes. Data shown are the relative abundance of the indicated mRNA derived from human or mouse cells normalized to that of GAPDH. Gene-specific primer sequences were previously described^42^.

### Co-immunoprecipitation, phosphorylation assays, and immunoblotting assays

Cells were lysed with lysis buffer (20 mM Tris–HCl, pH 7.5; 1% Nonidet P-40; 10 mM NaCl; 3 mM EDTA and 3 mM EGTA) containing complete protease inhibitors and 1% SDS, and denatured by heating for 10 min. The lysates were diluted with lysis buffer until the concentration of SDS was decreased to 0.1% and sonicated for 1 min, then the lysates were centrifugation at 16,000 × *g* for 15 min at 4 °C. The supernatants were immunoprecipitated with the indicated antibodies or anti-FLAG agarose beads. The beads were washed with cold lysis buffer for three times. The bound proteins were separated by SDS-PAGE, followed by immunoblotting analysis with the indicated antibodies.

### In vitro kinase assays

For in vitro kinase assays, anti-FLAG immunoprecipitates from asynchronous THP1 cells overexpressing FLAG-cGAS were washed three times with lysis buffer and resuspended in ice-cold kinase buffer (50 mM Tris–HCl pH 7.5, 10 mM MgCl_2_, 2 mM DTT, 10 μM ATP, 1 mM β-glycerophosphate, 0.1 mM Na_3_VO_4_). The immunoprecipitates were then incubated with purified CDK1-cyclin B (Life Technologies) pretreated with or without CDK1 inhibitor RO-3306 (10 μM) at 30 °C with constant shaking for 1 h. The reaction was stopped by SDS-PAGE loading buffer and heating to 95 °C for 5 min. The proteins were subjected to immunoblotting analysis with the indicated antibodies.

### In vitro cGAMP synthesis assays

For in vitro cGAMP synthesis assays, anti-FLAG immunoprecipitates from asynchronized or mitotic HT1080 cells overexpressing FLAG-cGAS were washed three times with lysis buffer and eluted by FLAG peptides. The eluted proteins were resuspended in ice-cold cGAMP synthesis buffer (20 mM Tris–HCl pH 7.5, 150 mM NaCl, 5 mM MgCl_2_, 1 mM DTT, 100 μM ATP, 100 μM GTP) containing HT-DNA and pyrophosphatase (0.25 U/ml). The reaction was then incubated at 37 °C for 1 h. The reaction was quenched by mixing with EDTA (25 mM) and the free-phosphate was detected by Malachite Green Phosphate Detection Kit (CST) following instructions of the manufacturer.

### Fluorescent confocal microscopy

For visualizing cGAS, the cells were fixed with 4% paraformaldehyde for 15 min, then permeablized for 15 min by incubation with PBS containing 0.1% Triton X-100. The cells were blocked with PBS containing 1% BSA and stained with the indicated antibodies and DAPI. Imaging of the cells was carried out using Zeiss confocal microscope under a ×63 (MLF) or ×100 (Raw264.7) oil objective.

### Time-lapse microscopy

H2B-RFP expressing HeLa cells were transfected with GFP-cGAS for 20 h before imaging with a Zeiss incubator microscope at 37 °C in 5% CO_2_. Images were acquired with a ×63 objective for 2 h.

### Measurement of cGAMP activity

Cells were transfected with the indicated synthetic dsDNA (3 μg/ml) for 4 h, and then cell extracts were prepared and heated at 95 °C to denature most proteins, which were removed by centrifugation. The supernatants containing cGAMP were delivered to digitonin-permeablized Raw264.7 at 37 °C for 30 min and then cells were further cultured with fresh medium for another 4 h before qPCR analysis.

### cGAMP quantification

HT1080 cells (1.5 × 10^8^) were left untreated or treated with RO-3306 (10 μM) for 30 min. Then cell extracts were prepared and heated at 95 °C to denature most proteins, which were removed by centrifugation. The supernatants containing cGAMP were measured by an Ultimate 3000 UHPLC Dionex (Sunnyvale, CA) coupled with a TSQ Quantiva (Thermo Fisher, Waltham, MA). The chromatography separation was performed on a Waters C18 column (100 × 2.1 mm i.d, 1.8 μm) at 40 °C. Selective reaction monitoring (SRM) and the appropriate product ions were chosen to quantify cGAMP. The ration is based on the standard.

### Statistical analysis

Quantitative data in histograms are shown as means ± s.d. Student’s *t*-test (unpaired, two-tailed) was used to analyze the differences of experimental and control groups. The number of asterisks represents the degree of significance with respect to *P* values. *P < 0.05, **P < 0.01, ***P < 0.001.

## Supplementary information

Supplementary Information

Supplementary Movie S1

## Data Availability

The data that support the findings of this study are available from the corresponding author upon request.

## Supplementary information

**Supplementary Information** accompanies the paper at (10.1038/s41421-020-0162-2).
