# Supplementary material for: Phosphorylation of cGAS by CDK1 impairs self-DNA sensing in mitosis
Source: Cell Discov. 2020 Apr 28;6:26. doi: 10.1038/s41421-020-0162-2 (PMC7186227; doi:10.1038/s41421-020-0162-2)
Supplement: Supplementary file 1 — Supplementary Information [file 41421_2020_162_MOESM1_ESM.pdf]

Supplementary Information

Zhong et al.

Supplementary Figures 1-3

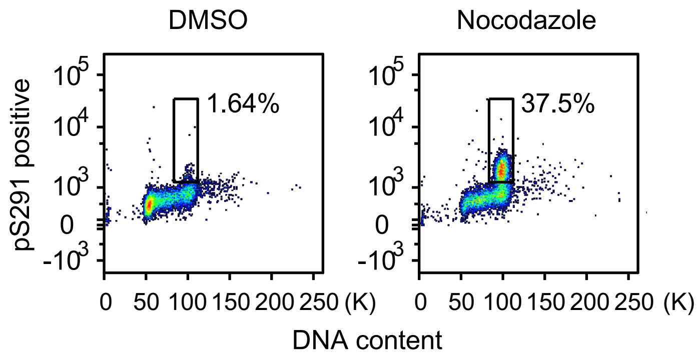

Supplementary Fig. S1. mcGAS is phosphorylated at S291 in mitotic Raw264.7 cells. The cells were untreated or treated with nocodazole (150 nM) for 8 h before FACS analysis with the indicated antibodies.

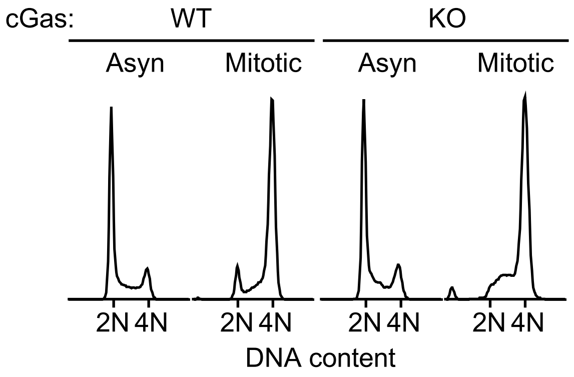

Supplementary Fig. S2. cGAS-deficiency does not affect cell cycle progression. *cGas*$^{+/+}$ and *cGas*$^{-/-}$ L929 cells were treated with nocodazole (300 nM) for 14 h and stained with Hoechst33342 before FACS analysis.

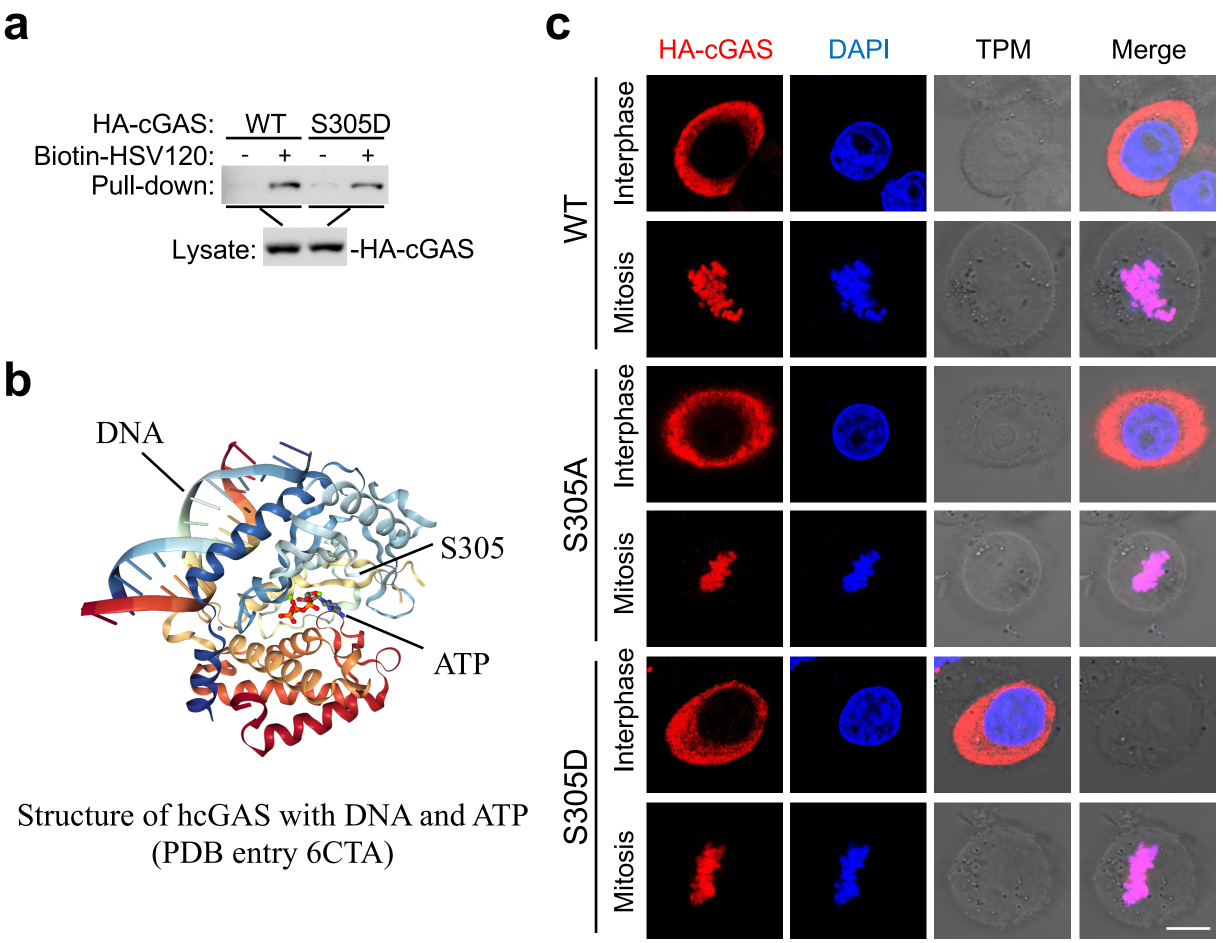

Supplementary Fig. S3. Functions of hcGAS S305 phosphorylation.

a Binding of hcGAS or hcGAS(S305D) to dsDNA. HEK293T cells were transfected with the indicated plasmids for 24 h followed by DNA pull-down and immunoblotting analysis with the indicated antibodies.

b S305 is located in the catalytic pocket of hcGAS. S305 is shown in the cGAS structure (PDB entry 6CTA).

c Localization of wild-type cGAS and its mutants. HeLa cells were transfected with the indicated HA-tagged expression plasmids for 36 h, then fixed and stained with anti-HA and DAPI before confocal microscopy. Scale bars, 10 μm.

Supplementary Movie S1. cGAS translocated into chromosomes upon NEBD .
